# Time critical diagnoses and transfers of patients with acute type A aortic dissection in the UK: national audit of current practice*

**DOI:** 10.1111/anae.16443

**Published:** 2024-10-07

**Authors:** Tom Gilbey, Benjamin Milne, Seema Agarwal, Siu‐Wai Choi, Simon Kendall, Joseph E. Arrowsmith, Gudrun Kunst, the ACTACC Linkperson Network, S. Cumming, P. Sasidharan, A. Idage, W. Abid, G. Namjoshi, F. Flynn, H. Singh, D. Hume, M. Hartley, A. Hill, M. Lewis, G. Ramalingam, E. Buckwell, R. Abel, M. Patteril, M. Steven, C. Rigg, M. Cross, A. Darbar, C. Yeo, S. Ripoll, J. Kendall, A. Hulme, C. Corredor, H. Parsons, A. Hodek, A. Chawla, H. Elmahdi, L. Szentgyorgi, K. Khan, S. Law, M. Prabhu, D. Woodward, A. Haines, P. Knowles, R. Jeeji, H. Taman, F. Husain, A. Allana, S. Sandys, D. Morrice, A. Meraglia

**Affiliations:** ^1^ Department of Anaesthesia King's College Hospital NHS Foundation Trust London UK; ^2^ Department of Anaesthesia Manchester Foundation Trust Manchester UK; ^3^ University of Manchester Manchester UK; ^4^ Department of Orthopaedics and Traumatology University of Hong Kong Hong Kong SAR; ^5^ NHS England North‐West Region UK; ^6^ Department of Anaesthesia Royal Papworth Hospital NHS Foundation Trust Cambridge UK; ^7^ School of Cardiovascular and Metabolic Medicine and Sciences King's College London London UK

**Keywords:** aortic dissection, cardiac anaesthesia, cardiothoracic critical care, transfer medicine

## Abstract

**Background:**

Type A aortic dissection repair is one of the most common emergency cardiac surgical procedures undertaken in the UK and has a high mortality. Early diagnosis and prompt surgery by an expert cardiac surgical team is crucial. Little is known about the patient's journey from first symptoms until surgery.

**Methods:**

The Association for Cardiothoracic Anaesthesia and Critical Care undertook a prospective national audit of the management of type A aortic dissection in the UK.

**Results:**

The details of 334 patients with type A aortic dissection were reported by 28 UK cardiac centres over 12 months. Median (IQR [range]) time from onset of symptoms until arrival in an emergency department was 2.3 (1.4–4.7 [0.1–491.6]) h. Median (IQR [range]) time between arrival in the emergency department and the start of surgery was 9.5 (6.1–18.2 [0.8–363.5]) h. Delays in diagnosis and transfers were reported in 158 (47.3%) patients. Fifty‐two patients (15.6%) had an initial misdiagnosis. The condition of 56 patients (16.8%) deteriorated clinically before arrival in the operating theatre. A medical doctor accompanied 50 patients (15.0%) during transfer. Sixty‐four patients (19.2%) died in hospital with 41 (12.3%) dying within the first 5 days after surgery.

**Conclusions:**

This audit provides a snapshot of current practice for patients with type A aortic dissection in the UK. Our findings show the acuity, clinical severity and vulnerability of patients with type A aortic dissection, and the deficits in the process of diagnosis and the quality of transfer. This audit demonstrates the need for the implementation of comprehensive, regionally governed, interdisciplinary medical management for every patient with type A aortic dissection.

## Introduction

Aortic dissection is a life‐threatening condition and an important cause of sudden death with a mortality rate of 7 per 100,000 cases in the UK [1]. Acute type A aortic dissection results from an intimal tear in the ascending aorta which normally requires urgent surgical repair [2]. Although many patients present with acute central chest pain which is at its worst at onset and radiating to the back, isolated limb ischaemia, abdominal pain or stroke as presenting complaints may make diagnosis challenging.

Around 80% of type A aortic dissections can be managed with replacement of the ascending aorta with an interposition graft. The remainder require specialist, multidisciplinary intervention and more complex surgery [2]. Up to 50% of patients with type A aortic dissection die before reaching hospital [3]. Untreated, cumulative mortality is estimated to be 1–2% of patients per hour following symptom onset, and up to 90% at 30 days. The prognosis is particularly poor for patients with organ dysfunction secondary to malperfusion [2, 3]. As such, type A aortic dissection is a cardiac surgical emergency and early diagnosis and prompt transfer to a specialist cardiothoracic centre will benefit patients.

The number of surgical procedures undertaken for type A aortic dissection in the UK has doubled during the last decade and now exceeds 500 cases per year [4]. Surgical repair has an 18% mortality burden, although there are outcome benefits over conservative management in appropriately selected patients [4, 5]. There is, however, a period between diagnosis and arrival at a surgical centre when patients may be exposed to treatment variations and unnecessary delays in care. Prompt and correct diagnosis permits swift surgical intervention, whereas a delayed diagnosis or misdiagnosis may delay surgery and potentially cause patient harm.

Very little is known about transfer procedures used, but the centrally planned health service and a relatively high population density in the UK offer a unique opportunity to assess this aspect of the pathway. We therefore decided to study transfer variables and peri‐operative treatment of patients with type A aortic dissection in detail by conducting a prospective national audit in UK cardiac surgical centres over 12 months with the Association for Cardiothoracic Anaesthesia and Critical Care (ACTACC) research collaboration and link‐person network (collaborators are listed in online Supporting Information Appendix S1). The ACTACC research collaboration has conducted previous national audits in relation to cardiac anaesthesia [6, 7].

Our primary aim was to provide a service evaluation of transfer logistics for patients with type A aortic dissection. Secondary aims included assessments of peri‐operative clinical variables and outcomes. This ACTACC audit provides a snapshot analysis of the journey of patients with type A aortic dissection from the onset of symptoms until hospital discharge, including current transfer standards and pre‐operative treatments.

## Methods

All UK cardiac surgical centres were approached by ACTACC for participation in the audit. As a service evaluation project, ethics committee approval was not sought (supported by the NHS Health Research Authority Decision Tool). Routine clinical governance procedures were followed by all participating centres.

The audit dataset was compiled in 2020 based on consensus by experienced clinicians in the fields of cardiac anaesthesia and intensive care medicine (ACTACC Scientific Committee) and cardiothoracic surgery (Society of Cardiothoracic Surgeons representative). These included nine cardiac anaesthetists and intensivists on the ACTACC board and the President of the Society of Cardiothoracic Surgeons. Additional insights were provided by other stakeholders, including subspecialty aortic communities and informed by work from the multi‐society ‘Think Aorta’ campaign (https://www.thinkaorta.net). The final dataset was subjected to a round of testing with local and national cases before launch. Data collection occurred via a secure online platform (SurveyMonkey, Palo Alto, CA, USA).

The final audit comprised 22 main question sets (online Supporting Information Appendix S2). Posters with quick response (QR) codes, paper copies of the form and online spreadsheets for data updates were sent to participating centres. We included patients with emergency/unplanned admissions to hospitals arriving in an operating theatre for repair of a type A aortic dissection. Patients with iatrogenic dissections during other cardiac procedures were not studied. Hypertension was defined as mean arterial pressure (MAP) ≥ 100 mmHg (> 130/85 mmHg) and hypotension as MAP < 65 mmHg [8].

An interval causing a delay between arrival in the emergency department and onset of surgery exceeding 60 min was defined as a named avoidable delay. In a qualitative approach, all responses of described delays were reviewed by two authors for their validity and only plausible delays were included in the final count. These delays were grouped into three categories: diagnosis‐related; transfer‐related; and delays at the receiving cardiac centre. Haemodynamic compromise was defined as a systolic BP < 90 mmHg at emergency department presentation or arrival in the operating theatre; neurological compromise was defined as Glasgow Coma Scale (GCS) < 12 at emergency department presentation or on arrival in the operating theatre or deterioration by > 3 points during transfer. Patients with incomplete clinical outcome results were excluded from the analyses.

Primary data were recorded in an Excel spreadsheet (Microsoft Corporation, Redmond, WA, USA) and assessed for normality using the D'Agostino‐Pearson omnibus normality test using GraphPad Prism version 7.04 (GraphPad Software, Inc.) Statistical analyses were done using SPSS v25 (SPSS, Armonk, NY) with Student's t‐test, Mann–Whitney U test and chi‐squared test used according to type of data and normality of data distribution. Logistic regression analysis was used to investigate variables and their ability to predict mortality. A p value of < 0.05 was considered statistically significant.

## Results

**Figure 1:**
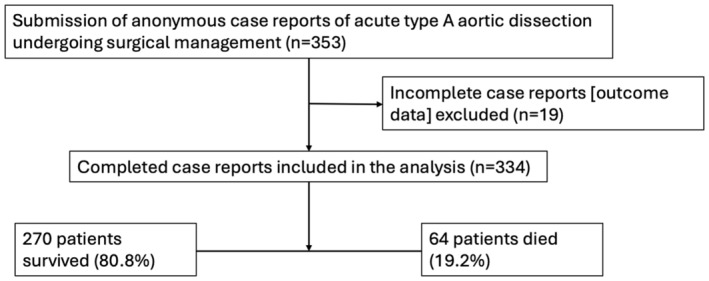
Study flow diagram of patients reported, included and analysed.

Twenty‐eight UK cardiac surgical units submitted patient data during the 12‐month study period which started on 1 May 2021. Of the 353 case reports submitted, 19 had incomplete follow‐up data and were excluded from the analyses (Fig. 1). Reporting and caseloads numbers at the 28 centres were variable; the median (IQR [range]) of cases reported was 10 (4–16 [1–39]) per centre. Twenty centres (71.4%) reported ≥ 5 cases, whereas eight centres (28.6%) reported < 5 cases.

The pre‐operative characteristics of patients with type A aortic dissection and transfer times in surviving and non‐surviving patients are shown in Table 1. The mean (SD) age of the cohort was 62 (13.5) y with 208 male patients (62.3%). Median (IQR [range]) time from onset of symptoms until arrival in an emergency department was 2.3 (1.4–4.7 [0.1–491.6]) h and from arrival in an emergency department until the beginning of surgery 9.5 (6.1–18.2 [0.8–363.5]) h, resulting in a time from onset of symptoms until the start of surgery of 12.5 (9.0–26.6 [1.2–538.1]) h. Time between arrival at the cardiac centre and the start of surgery was 2.4 (1.4–4.8 [0.2–363.5]) h. Twelve patients (3.6%) presented and were admitted directly to a cardiac centre.

**Table 1:** Pre‐operative characteristics and transfer times in patients with acute type A aortic dissection repair. Values are mean (SD), number (proportion) or median (IQR [range]).

	All patients	Survivors	Non‐survivors
	n = 334	n = 270	n = 64
Age; y	62 (13.5)	62 (13.5)	64 (13.7)
Sex; male	208 (62.3%)	166 (61.5%)	42 (65.6%)
Symptom onset to ED attendance; min*	137 (85–282 [4–29,494])	138 (85–360 [10–29,494])	131 (98–203 [4–3840])
ED attendance to start of surgery; min^†^	570 (364–1091 [50–21,812])	575 (387–1238 [50–21,812])	531 (349–830 [60–9350])
Symptom onset to start of surgery; min^‡^	750 (539–1595 [74–32,286])	811 (542–1845 [120–32,286])	688 (480–996 [74–9480])
ED attendance to arrival at cardiac centre; min^§^	373 (186–735 [0–18,798])	392 (211–833 [0–18,798])	327 (19–480 [0–9020])
Arrival at cardiac centre to start of surgery; min^¶^	143 (81–288 [9–21,812])	145 (90–290 [9–21,812])	122 (63–237 [20–8095])

ED, emergency department; *, n = 39 missing; ^†^, n = 23 missing; ^‡^, n = 25 missing; ^§^, n = 24 missing; ^¶^, n = 4 missing.

On arrival in the emergency department, MAP measurements were available in 284 patients, with a mean (SD) of 93 (28) mmHg (Table 2). In total, 301 patients had a GCS of 15 (90.1%), 17 (5.1%) a reduced GCS of 13–14 and 11 (3.3%) a GCS of ≤ 12 (Table 2). In 319 patients (95.5%), the diagnosis of type A aortic dissection was made at the first hospital of admission using computed tomographic (CT) imaging. In addition, 113 (33.8%) patients also had echocardiographic assessment.

**Table 2:** Diagnosis, transfer and clinical management before surgery of patients with acute type A aortic dissection. Values are mean (SD) and number (proportion).

	All patients	Survivors	Non‐survivors
	n = 334	n = 270	n = 64
**On emergency department presentation**			
Mean arterial pressure; mmHg*	93 (28.2)	94 (29.7)	87 (19.8)
Glasgow Coma Scale^†^			
15	301 (90.1%)	250 (92.6%)	51 (79.7%)
13–14	17 (5.1%)	11 (4.1%)	6 (9.4%)
9–12	4 (1.2%)	3 (1.1%)	1 (1.6%)
4–8	4 (1.2%)	0	4 (6.3%)
3	3 (0.9%)	2 (0.7%)	1 (1.6%)
Misdiagnosis			
None	282 (84.4%)	227 (84.1%)	55 (85.9%)
Acute coronary syndrome	27 (8.1%)	25 (9.3%)	2 (3.1%)
Cerebrovascular accident (alone)	6 (1.8%)	1 (0.4%)	5 (7.8%)
Pulmonary embolus	12 (3.6%)	12 (4.4%)	0
Non‐type A aortic dissection	3 (0.9%)	2 (0.7%)	1 (1.6%)
Musculoskeletal diagnosis	2 (0.6%)	2 (0.7%)	0
Gastritis	2 (0.6%)	1 (0.4%)	1 (1.6%)
**During transfer**			
Deterioration	56 (16.8%)	32 (9.6%)	24 (37.5%)
Neurological	10 (3.0%)	5 (1.9%)	5 (7.8%)
Haemodynamic	26 (7.8%)	20 (7.4%)	6 (9.4%)
Cardiac arrest	3 (0.9%)	2 (0.7%)	1 (1.6%)
Other malperfusion	6 (1.8%)	1 (0.4%)	5 (7.8%)
Pain	2 (0.6%)	1 (0.4%)	1 (1.6%)
Hypertension	1 (0.3%)	0	1 (1.6%)
**On arrival at cardiac centre**			
Monitoring^§^			
ECG	278 (83.2%)	220 (65.9%)	58 (90.6%)
Oxygen saturation	260 (77.8%)	204 (75.6%)	56 (87.5%)
Capnography	10 (3.0%)	4 (1.5%)	6 (9.4%)
Non‐invasive blood pressure	236 (70.7%)	191 (70.7%)	45 (70.3%)
Invasive blood pressure	61 (18.2%)	48 (17.8%)	13 (20.3%)
Central venous catheter	12 (3.6%)	5 (1.9%)	7 (10.9%)
Therapies instituted^¶^			
Supplemental oxygen	134 (40.1%)	98 (36.3%)	26 (40.6%)
Mechanical ventilation	7 (2.1%)	1 (0.4%)	6 (9.4%)
Labetalol infusion	79 (23.7%)	65 (24.1%)	14 (21.9%)
Glyceryl trinitrate infusion	20 (6.0%)	19 (7.0%)	1 (1.6%)
Inotrope or vasopressor	11 (3.3%)	8 (3.0%)	3 (4.7%)
Analgesia	5 (1.5%)	2 (0.7%)	3 (4.7%)
None	27 (8.1%)	25 (9.3%)	2 (3.1%)
**On arrival in operating theatre**			
Glasgow Coma Scale**			
15	292 (87.4%)	248 (91.9%)	44 (68.8%)
13–14	22 (6.6%)	13 (4.8%)	9 (14.1%)
9–12	5 (1.5%)	4 (1.5%)	1 (1.6%)
4–8	2 (0.6%)	0	2 (3.1%)
3	10 (3.0%)	3 (1.1%)	7 (10.9%)
Mean arterial pressure; mmHg††	85 (21.8)	87 (22.1)	79 (19.3)

*, n = 50 missing; ^†^, n = 5 missing; ^§^, n = 1 missing; ^¶^, n = 15 missing;**, n = 3 missing; ^††^, n = 13 missing.

Delays between emergency department arrival and the start of surgery were described in 158 patients (47.3%). These included: delayed diagnosis (n = 86, 25.7%); transfer‐related (n = 38, 11.4%); combined diagnosis and transfer‐related (n = 24, 7.2%); and logistic issues at the receiving centre (n = 10, 3.0%). The latter category included a lack of available operating theatres, a lack of blood products or consent issues. In 52 patients (15.6%), the delay in diagnosis was associated with a misdiagnosis (Table 2). Of patients with an initially incorrect diagnosis, the majority were diagnosed with acute coronary syndrome (n = 27, 8.1%); pulmonary embolus (n = 12, 3.6%); or cerebrovascular accident (n = 6, 1.8%) (Table 2). Examples of free‐entry comments by ACTACC link‐persons related to diagnostic and transfer delays are presented in Box 1. Whereas 304 patients (91.0%) were accepted by the first cardiac surgical centre to which they were referred, 30 patients (9.0%) could not be accepted (Table 3).

Box 1 **Free entry comments by ACTACC link‐persons regarding diagnostic and transfer delays.**

**Diagnosis**

- Delay in diagnosis from the emergency department, patient was sent home, collapsed in the car on his way home. Presented to emergency department on 27th with chest pain. Discharged home. Returned again on 30th with hypotension. Arrest call in ward for severe hypotension; transferred to ICU in local hospital; CT; diagnosed aortic dissection; transferred to cardiac centre.
- > 6 h to consider aortic dissection and order CT scan.
- First admitted to hospital with chest pain 01/04 at 10.30 and discharged. Second admission to hospital via ambulance 01/04 at 20.20. Diagnosis made and patient transferred to operating hospital.
- Needed CT head and carotid imaging. Presented with stroke‐like symptoms.
- Delay in diagnosis. Patient discharged home first time with a diagnosis of acute gastritis. Patient re‐presented 12 h later with dyspnoea and cardiogenic shock.
- 3 days to diagnose, was investigated for gastrointestinal bleed, found to have different pulses at oesophagogastroduodenoscopy.
- 15 days! Delayed diagnosis, eventually via privately requested elective CT scan.

**Transfer logistics**

- The first hospital was itself a cardiac centre, which is able to perform aortic surgery, but aortic surgeons were already operating and there was no theatre/staff capacity available. > 5 h delay between first hospital and arrival at second cardiac centre.
- Ambulance available for transfer at 22.00 despite referral at 19.30.
- Decision to operate made at 14.30. Transfer not initiated until 19.30. Lack of communication between teams.
- Delay in transfer – no ambulance.

**Table 3:** Details of cardiac centre referral for patients with acute type A aortic dissection. Values are number (proportion).

	All patients	Survivors	Non‐survivors
	n = 334	n = 270	n = 64
Accepted by first centre	304 (91.0%)	249 (92.2%)	55 (85.9%)
Reason for refusal by first centre			
Critical care capacity	3 (0.9%)	3 (1.1%)	0
Operating theatre capacity	8 (2.4%)	6 (2.2%)	2 (3.1%)
Staffing issues	1 (0.3%)	1 (0.4%)	0
Inadequate surgical expertise	5 (1.5%)	2 (0.7%)	3 (4.7%)
Patient not for surgery (as per that centre)	2 (0.6%)	1 (0.4%)	1 (1.6%)
Centre not accepting referrals	4 (1.2%)	4 (1.5%)	0
Not specified	7 (2.1%)	4 (1.5%)	3 (4.7%)

The majority of patients (n = 284, 85.0%) were transferred by a transfer team without a medical doctor. The members of the transfer teams are shown in Table 4.

**Table 4:** Transfer team constitution for patients with acute type A aortic dissection. Values are number (proportion).

Team members	All patients n=334	Survivors n=270	Non‐ survivors n=64
Nurse	90 (26.9%)	80 (29.6%)	10 (15.6%)
Ambulance technician	101 (30.2%)	80 (29.6%)	21 (32.8%)
Paramedic	195 (58.4%)	153 (56.7%)	42 (65.6%)
Non‐airway doctor	24 (7.2%)	19 (7.0%)	5 (7.8%)
Airway‐trained doctor	30 (9.0%)	22 (8.1%)	8 (12.5%)
Any doctor	50 (15.0%)	39 (14.4%)	11 (17.2%)
Regional retrieval team	6 (1.8%)	6 (2.2%)	0
Grade of medical escort^††^			
Senior house officer	5 (1.5%)	3 (1.1%)	2 (3.1%)
Junior registrar	14 (4.2%)	14 (5.2%)	0
Senior registrar	19 (5.7%)	15 (5.6%)	4 (6.3%)
Speciality or Associate Specialist	2 (0.6%)	0	2 (3.1%)
Consultant	10 (3.0%)	7 (2.6%)	3 (4.7%)
No doctor	269 (80.5%)	216 (80%)	53 (82.8%)

^††^n = 15 missing.

The condition of 56 patients (17%) deteriorated before arrival in an operating theatre. These included haemodynamic deterioration or cardiac tamponade in 26 patients (7.8%); neurological deterioration in 10 (3.0%); abdominal organ malperfusion of gut or kidneys in six (1.8%); and cardiac arrest in 3 (0.9%) (Table 2). Thirteen (23.2%) of the deteriorating patients had a medical escort during their transfer and 43 (76.8%) deteriorated clinically without a doctor present.

The treatment of patients before arrival at a cardiac centre is shown in Table 2. This included supplemental oxygen in 134 patients (40.1%); labetalol administration in 79 (23.7%); glyceryl trinitrate infusion in 20 (6.0%); and vasopressor or inotrope infusions in 11 (3.3%). Details of invasive and non‐invasive monitoring of patients on arrival in the cardiac centre are shown in Table 2, with 236 patients (70.7%) receiving minimal non‐invasive monitoring consisting of ECG in 278 (83.2%); oxygen saturation in 260 (77.8%); and intermittent non‐invasive blood pressure monitoring in 236 (70.7%). Neurological deterioration on arrival in the operating theatre was associated with significantly increased mortality (OR (95%CI) 9.6 (3.2–29.1), p < 0.001). The association of haemodynamic deterioration and mortality failed to reach statistical significance (OR (95%CI) 2.3 (1.0–5.1), p = 0.05).

Intra‐operative surgical and anaesthetic techniques are summarised in Table 5. Patients having type A aortic dissection repair and concomitant coronary artery bypass graft surgery (n = 31) had a significantly increased mortality (11 patients (17%) died vs. 20 patients (7.5%) who survived, p = 0.03) (Table 5). Patients who died in hospital had a significantly lower minimum peak temperature during cardiopulmonary bypass when compared with survivors (median (IQR [range]) 19.3 (18.0–22.7 [15–36])°C vs. 21.7 (19.0–25.0 [12–35.3])°C, p = 0.001), longer periods of deep hypothermic cardiac arrest (median (IQR [range]) 38 (25–63 [5–116]) min vs. 30 (20–46 [2–201]) min, p = 0.039) and received a higher number of packed red cells during surgery (median (IQR [range]) 2 (0–5 [0–17]) units vs. 1 (0–3 [0–21]) units, p = 0.02).

**Table 5:** Intra‐operative management of patients with acute type A aortic dissection. Values are number (proportion) or median (IQR [range]).

	All patients	Survived	Non‐survivors
	n = 334	n = 270	n = 64
**Surgical procedure**			
Aortic valve replacement	90 (26.9%)	77 (28.5%)	13 (20.3%)
Aortic procedure*			
Aortic root repair	1 (0.3%)	1 (0.4%)	0
Aortic root replacement	24 (7.2%)	17 (6.3%)	7 (10.9%)
Ascending aorta replacement	137 (41.0%)	118 (43.7%)	19 (29.7%)
Hemi‐arch replacement	119 (35.6%)	102 (37.8%)	17 (26.6%)
Aortic arch replacement	13 (3.9%)	9 (3.3%)	4 (6.3%)
Frozen elephant trunk	28 (8.4%)	20 (7.4%)	8 (12.5%)
No procedure/died on table	7 (2.1%)	0	7 (10.9%)
Additional coronary artery bypass graft	31 (9.3%)	20 (7.4%)	11 (17.2%)
Re‐do procedure	8 (2.4%)	4 (1.5%)	4 (6.3%)
Delayed closure	8 (2.4%)	7 (2.6%)	1 (1.6%)
Chest re‐opened	11 (3.3%)	7 (2.6%)	4 (6.3%)
**Intra‐operative management**			
Aortic cross‐clamp time; min	122 (86–173 [31–427])	121 (85–167 [31–427])	133.5 (91–182 [58–377])
Use of deep hypothermic circulatory arrest	238 (71.3%)	197 (73.0%)	41 (64.1%)
Duration; min	30 (21–49 [2–201])	30 (20–46 [2–201])	38 (25–63 [5–116])
Lowest temperature; °C	21.0 (18.5–24.3 [12.0–36.0])	21.7 (19.0–25.0 [12.0–35.3])	19.3 (18.0–22.7 [15.0–36.0])
Blood products			
Red blood cells; units	2 (0–5 [0–21])	1 (0–3 [0–21])	2 (0–5 [0–17])
Fresh frozen plasma; units	3 (1–4 [0–34])	3 (2–4 [0–19])	4 (0–4 [0–34])
Platelets; pools	2 (1–2 [0–9])	2 (1–2 [0–6])	2 (1–3 [0–9])
Cryoprecipitate; pools	2 (0–2 [0–20])	2 (0–2 [0–20])	0 (0–2 [0–12])
Prothrombin complex concentrate; units	0 (0–1000 [0–4000])	0 (0–1000 [0–4000])	0 (0–1000 [0–3000])
Fibrinogen concentrate; mg	0 (0–0 [0–17,000])	0 (0–0 [0–5000])	0 (0–0 [0–17,000])

* n = 5 missing.

**Figure 2:**
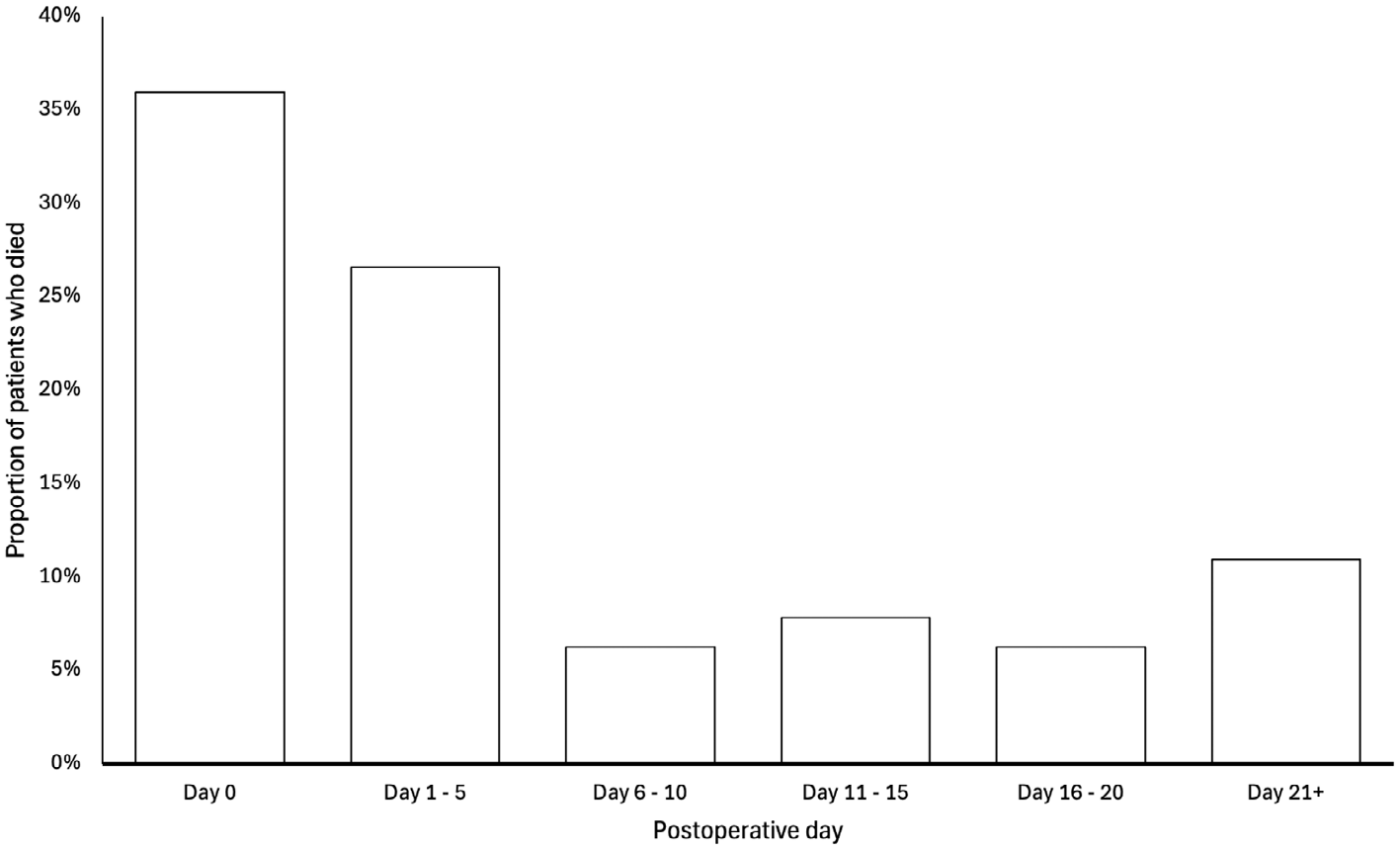
Postoperative mortality of patients with acute type A aortic dissection by postoperative day.

Sixty‐four patients (19.2%) died and 41 of these (64.1%) died on the day of surgery or within five days postoperatively (Fig. 2). The 12 patients (3.6%) that presented directly to a cardiac centre all survived; the time to transfer was verified as zero in these patients. The median (IQR [range]) day of postoperative death was 2 (0–11 [0–106]) and median duration of stay of surviving patients was 15 (11–27 [2–147]) days in hospital. Of the surviving cohort of patients, only 46 (17.0%) had a duration of hospital stay ≤ 10 days.

## Discussion

This 12‐month ACTACC audit provides a snapshot of transfer logistics in patients presenting with type A aortic dissection in the UK. We found significant transfer and diagnostic delays before surgery in 158 (47.3%) patients, with 52 (15.6%) having been initially misdiagnosed. Fifty‐six patients (16.8%) deteriorated before their arrival in an operating theatre. In total, 284 patients (85%) did not have a doctor present during their transfer and minimal monitoring standards (including ECG, non‐invasive blood pressure and oxygen saturation) were missing in 98 patients (29.3%). In summary, our results show the severity and acuity of type A aortic dissection syndrome, but with gaps in medical care which should be addressed by timely diagnoses and time‐efficient and safer transfers.

The characteristics and mortality of patients with type A aortic dissection presented here mirrors a previously described case series of patients with a median age of 65 y, underrepresentation of female patients (37.7%) and a mortality of 19.2% [4].

Immediate surgery is the gold standard for most patients with type A aortic dissection [9]. Delayed diagnosis and misdiagnosis can be explained by the fact that the symptoms and signs of type A aortic dissection are often non‐specific and may vary or mimic other pathologies such as acute coronary syndrome, pulmonary embolus or stroke [2]. Therefore, a high degree of suspicion is required for early recognition of dissection in patients presenting with severe chest pain. This can only be achieved by implementation of regular education and training programmes for all emergency department staff and diagnostic teams, led by regional multidisciplinary type A aortic dissection teams. In the south of England this has recently been addressed by implementation of a supra‐regional aortic dissection standard operating procedure, which illustrates diagnostic pathways and an Aortic Dissection Detection Risk Score for medical teams [10]. Furthermore, the global ‘Think Aorta’ campaign has raised awareness within hospitals, emergency department teams and patient groups with the expressed aim of improving the diagnosis of type A aortic dissection worldwide. Importantly, this campaign also addresses patients and laypersons, with the result that they will be prompted by the campaign to present as early as possible at hospitals for an exclusion of type A aortic dissection if they experience severe chest pain.

The large variation in transfer times and transfer delays indicates that regional governance for referral pathways are lacking. Whereas longer transfer times due to long distances in non‐urban settings are unavoidable, unavailability of ambulances or crews or delayed departure must be avoided by improved resources and regional governance, with prioritisation of patients with type A aortic dissection. A high proportion of patients were managed by the first cardiac centre to which they were referred, however, 23 patients (6.9%) were refused due to operating theatre/critical care capacity or a lack of surgical expertise. This logistic shortfall in communication between transfer teams and treatment teams should be addressed by regionally agreed and published emergency referral pathways co‐ordinated by regional multidisciplinary type A aortic dissection teams. Furthermore, in order to provide a simple and rapid referral, there should be a regional 24/7 single point of contact for every emergency department and for transfer teams directing every patient along appropriate pathways [11].

The recently published UK Aortic Dissection Toolkit suggests regional key elements such as regional governance; coordination through regional multidisciplinary teams and meetings; regional rotas; and timely and reliable image transfer [11]. Implementation of these tools has now started in the UK, with national and regional working groups coordinating and implementing more regional governance structures. The multidisciplinary South England Aortic Dissection Network addresses this with a standard operating procedure for the management of all acute aortic syndromes, including type A aortic dissection, with the aim of minimising the risks of delayed diagnosis, facilitating smooth referrals and offering expeditious and appropriate therapies for all patients [10]. National interdisciplinary recommendations on the interhospital transfer of patients with acute aortic syndrome were recently published, in recognition of the need for standardised care of these patients. This document contributes to safer transfer of patients with type A aortic dissection [12].

Fifty‐six patients (16.8%) in this national audit presented with worsening organ function during their transfer and only 13 (23.2%) of these clinically deteriorating patients had a medical escort. Critical care transfer teams are necessary to care for these potentially unstable patients. Under their care, monitoring will be appropriate with basic haemodynamic monitoring for every patient and necessary treatment (with blood pressure control and analgesia) may be facilitated during the entire transfer period. Furthermore, haemodynamic and neurological deterioration can be appropriately managed medically. Safe transfers are also addressed by the UK Aortic Dissection Toolkit, recommending transfer by the regional Adult Critical Care Transfer services if possible [11]. This is particularly important, as the results of this audit show that clinical deterioration, such as neurological compromise (i.e. reduction of GCS) is associated with poorer outcomes.

Interestingly, a greater interval between the onset of symptoms and the start of surgery was not associated with higher hospital mortality. On the contrary, the timeline from the onset of symptoms to surgery was significantly shorter in patients who died after surgery in hospital. One explanation for these results could be that the cohort of patients in this audit did not include the number of patients (usually 50%) who die before their arrival in hospital, i.e. very sick and decompensated patients who may have had extended transfer times but died before arrival [3]. These patients are not reported nationally and in order to better assess how many patients with type A aortic dissection die before they reach hospital there should be national reporting of all sudden deaths with a post‐mortem diagnosis of type A aortic dissection. The highest risk for decompensation is usually in the initial hours after symptom onset. Another explanation for shorter transfer times of patients who died after surgery may be that sicker patients with overall poorer outcomes already presented with signs of clinical deterioration, such as lower GCS scores; faster transfers are likely to have been facilitated under these circumstances. However, due to the severity and acuity of type A aortic dissection, every patient should receive the fastest and safest transfer possible – not just those who are clinically unstable. Finally, the audit dataset did not distinguish between ‘emergency’ or ‘unplanned’ admissions at the cardiac centres. In particular, the times between arrival at the cardiac centre to start of surgery (median 2.4 h) may have been extended for some patients, e.g. those who were admitted to the critical care unit first, but not for others considered as potentially higher‐risk emergency patients who went straight to an operating theatre. In addition, most cardiac centres do not routinely have an empty emergency theatre and, if the patient arrived at a time when other cases were ongoing, there may have been additional delay.

Strengths of this audit include prospective data collection, as variables such as transfer timelines and quality of transfer are normally not accessible in retrospective registries such as the National Adult Cardiac Surgery Audit database. The prospective study design was only possible through the ACTACC link‐person network in the UK.

One of the limitations of this audit is bias due to ignorance of the total number of patients developing type A aortic dissection in the UK annually. Underreporting was caused by not all UK centres contributing to the audit (28 of 33 centres, 84.8%) and eligible patients not being reported by contributing centres. The most recently published annual number of type A aortic dissection patients in the UK was 541 in 2018 [4]. The National Adult Cardiac Surgery Audit reporting framework (run by the National Institute for Cardiovascular Outcomes Research) has recorded 535 cases of acute type A aortic dissection being managed surgically within the 12‐month ACTACC audit period. Therefore, our recruitment number of 334 patients included 62% of all UK annual cases. Previous reports indicate that response rates of at least 50–60% minimise the risk of non‐response bias [13] and therefore the number of reported cases in this audit appears to be sufficient to reduce non‐response bias satisfactorily. Apart from non‐response bias, recall bias and misinterpretations of responses are also limitations of this prospective audit dataset, which must be considered in the interpretation of the data.

Five cases per year is the minimal number of type A aortic dissection repairs per surgeon required to minimise the risk of mortality [4]. In our cohort, there were eight centres with fewer than five reported cases, which could reflect underreporting, or a true low number. Shared type A aortic dissection services or rotating cover between two or more centres would also reduce the number of patients in one single centre. It is a limitation of this audit that we cannot report the exact reasons for the low number of patients in these eight centres.

We did not include specific questions about travel times from referral hospitals until arrival in the cardiac centre; times from ambulance call to ambulance arrival; type of ambulance calls; and category of emergency ambulance transfer, which would have been important information to clarify further details about the ambulance transferral. These questions should be addressed in future audits and service evaluations.

Patients presenting with type A aortic dissection are one of the most acute and vulnerable patient groups and can be compared with other groups of high‐risk patients in need of urgent transfers, e.g. trauma patients. However, whereas regional trauma care has been prioritised, structured and implemented in trauma centres, analogous appropriate care of patients with type A aortic dissection now requires addressing. Recommendations by the authors to address the described gaps of management and medical care of patients with type A aortic dissection in this national ACTACC audit and are in‐line with the UK Aortic Dissection Toolkit [11] and are shown in Box 2.

Box 2 **Recommendations to address the described gaps of management and medical care of patients with type A aortic dissection.**

- Education programmes for diagnosis of type A aortic dissection for emergency department staff and diagnostic teams led by regional multidisciplinary type A aortic dissection teams will be needed for regular training and education.
- Regional multidisciplinary type A aortic dissection teams are necessary to co‐ordinate and publish emergency referral pathways.
- There should be a regional 24/7 single point of acute aortic syndrome contact for every emergency department and for transfer teams directing patients to the appropriate cardiac surgical centre to provide smooth and rapid transfers.
- Critical care transfer teams will be needed for the safe transfer of all patients with type A aortic dissection.

In summary, this prospective ACTACC national audit shows that many patients with type A aortic dissection have emergency surgery delayed because of holdups during diagnosis and transfer. Outcomes of patients with type A aortic dissection should be improved by prompt diagnoses and by regionally managed critical care patient transfer pathways, resulting in comprehensive and appropriate care for all patients with type A aortic dissection.

## Supporting information


**Appendix S1.** ACTACC Link‐Person Network Collaborators.

**Appendix S2.** Audit questionnaire.
