# Supplementary material for: Time critical diagnoses and transfers of patients with acute type A aortic dissection in the UK: national audit of current practice*
Source: Anaesthesia. 2024 Oct 7;80(3):248–58. doi: 10.1111/anae.16443 (PMC11825214; doi:10.1111/anae.16443)
Supplement: Supplementary file 1 — Appendix S1. ACTACC Link‐Person Network Collaborators. [file ANAE-80-248-s001.docx]

**Appendix S1** ACTACC Link-Person Network Collaborators

*Local coordinators & collaborating authors (Hospital):*

Cumming S, Sasidharan P (Aberdeen Royal Infirmary); Idage A, Abid W, Namjoshi G (Essex Cardiothoracic Centre); Flynn F (Royal Victoria Hospital, Belfast); Singh H, Hume D (Queen Elizabeth Hospital, Birmingham); Hartley M (Lancashire Cardiac Centre, Blackpool); Hill A (Royal Sussex County Hospital, Brighton); Lewis M (Bristol Royal Infirmary); Ramalingam G (Papworth Hospital, Cambridge); Buckwell E, Abel R (University Hospital of Wales, Cardiff); Patteril M (University Hospitals Coventry and Warwickshire); Steven M (Golden Jubilee National Hospital, Glasgow); Rigg C (Castle Hill Hospital, Hull); Cross M (Yorkshire Heart Centre, Leeds); Darbar A (Glenfield Hospital, Leicester); Yeo C, Ripoll S, Kendall J (Liverpool Heart and Chest Hospital); Hulme A, Corredor C (Barts Heart Centre, London); Parsons H, Hodek A (Harefield Hospital, London); Chawla A (St Thomas’ Hospital, London); Elmahdi H (Manchester Royal Infirmary); Szentgyorgi L (Wythenshawe Hospital, Manchester); Khan K, Law S (James Cook University Hospital, Middlesborough); Prabhu M (Freeman Hospital, Newcastle); Woodward D, Haines A, P. Knowles P (Northern General Hospital, Sheffield); Jeeji R (University Hospital North Midlands, Stoke on Trent); Taman H, Husain F (Morriston Hospital, Swansea); Allana A, Sandys S (University Hospital Southampton); Morrice D, Meraglia A (New Cross Hospital, Wolverhampton).
