# Supplementary material for: Time critical diagnoses and transfers of patients with acute type A aortic dissection in the UK: national audit of current practice*
Source: Anaesthesia. 2024 Oct 7;80(3):248–58. doi: 10.1111/anae.16443 (PMC11825214; doi:10.1111/anae.16443)
Supplement: Supplementary file 2 — Appendix S2. Audit questionnaire. [file ANAE-80-248-s002.docx]

**Appendix S2** Audit Questionnaire

1 Patient age

2 Sex

3 GCS on arrival in theatre?

4 BP on arrival in theatre?

5 HR on arrival in theatre?

6 Monitoring on arrival at cardiac centre

ECG

Sats

Non-invasive BP

invasive blood pressure

central line

intubated

other __________

7 Any of the following medications on arrival – please tick if yes

on oxygen mask

labetalol

gtn

propofol

other _____________

8 Transferral team at arrival at cardiac centre

health care worker / nurse

medically trained doctor

anaesthetist

if doctor what grade ______

9 Onset of initial symptoms time: (__:__) and date (day/month/year)

10 Admission to first hospital (A&E): approximate time (__:__) and date (day/month/year)

11 Investigations for diagnosis at first centre

CT

Echo

Other ___________

12 Arrival at cardiac centre hospital: time (__:__) and date (day/month/year)

13 Start of surgery: time (__:__) and date (day/month/year)

14 Type of surgery (several answers possible)

root replacement

ascending aorta replacement

arch replacement

concomitant CABG procedure

concomitant valve procedure

concomitant other procedure

first time procedure

re-do procedure

other _______________

15 Intraoperative use of (please tick if this technique was used, several answers possible)

tranexamic acid

aprotinin

cerebral perfusion

cerebral oximetry

DHCA

if DHCA used duration of DHCA ______min

16 Lowest intraop temperature (degrees Celsius)

________

17 Intraoperative cross clamp time (min)

________

18 Total blood products given in theatre (PRC, FFP, platelets, other) please list

________

19 Were there any significant delays (>1hr) during the patient’s journey between onset of symptoms and admission to theatres

Yes

No

If Yes which one

20 Postop: did patients die in hospital

Yes

No

If YES which postop day _____

21 iI patient survived

length of ICU stay

22 If patient survived

length of hospital stay
